# Trajectories in nanotechnology: embracing complexity, seeking analogies

**DOI:** 10.1007/s13346-020-00877-3

**Published:** 2021-01-28

**Authors:** Alexander T. Florence

**Affiliations:** UCL School of Pharmacy, London, WC1 UK

**Keywords:** Colloids, Nanoparticle behaviour, Biological-physical interfaces, Complexity, Analogies

## Abstract

This account comprises personal reflections on the field of nanosystems primarily designed for the delivery of biologically active agents. It emphasises the colloidal nature of nanoparticles obeying the same physical laws that dictate the behaviour of disperse systems. Research reveals not only intrinsic complexities but a variety of possible trajectories in vivo and ex vivo, issues of stability, interactions and behaviour in a range of often constrained environments. Such are the variations in the chemical and physical nature of the nanosystems and the active agents they carry, their putative “targets” and the many biological systems and models in which they are employed, it is not possible to generalise. Stochastic events may exclude precise prediction or extrapolation of outcomes, but embracing and studying complexity lead to new insights, often aided by consideration of analogies in cognate areas. This is part of the process of illumination. Unexpected results provide the true essence and excitement of scientific endeavour. Simplification is perhaps its antithesis.

## Introduction

“We cannot make facts: our wishing cannot change them: we must use them”. - John Henry Newman (1801–1890)

Our life as individuals can be described as a trajectory. Some events, which we may deem positive or negative, are the result of chance or coincidence. The author’s route began with research on nonionic surfactants with micellar diameters *circa* 50 Å, nanosystems decades before the advent of “nano” terminology. There followed a range of colloidal systems and then nanosystems, some by chance through interactions with industry, others by learning from failed attempts to harness the specificity of natural targeting systems such as low density lipoproteins [1]. The trajectories of nanosystems (administered often with the hope of them releasing their drug load at specific biological target endpoints) are by nature unpredictable, but much can be learned in the process. Nanoparticles are affected by the vagaries of stochastic events and by the nature and structure of vessels, cells and targets. Injected intravenously, they traverse multiple bifurcations en route*,* only some leading to coupling with desired targets. The laws of physics as well as biological realities impinge upon their performance as drug carriers, so it is imperative to confront and identify the nature of the complexities of the interactions of nanosystems with their biological environments. Both Newman’s sentiments cited above and scientific rigour insist that we do not cite mechanisms of action without proof that these are applicable to one’s particular system. Often glibly cited as a means of delivery is the enhanced permeation and retention (EPR) effect first elaborated by Maeda [2], but as the exponent of the effect, he has made it clear that it *does not* apply to every system or under all conditions. We must apply forensic rigour to all our findings.

## Obstructions

There are many obstructions to any “guided” or “targeted” movement of nanoparticles, in addition to their behaviour, say, in multi-bifurcated blood vessels and in cells. These include the aptly named *obstruction effect* [3] when particle diffusion is inhibited by objects in their path such as polymers [4] or actin threads in cells [5]. *Brownian motion* also influences the movement and interaction of particles in confined spaces such as the extracellular space of tumours or at the sites of both particle-ligand and drug-ligand receptor interactions. One might imagine that retarded particle movement would negatively affect the latter, but Guigas and Weiss [6] have pointed out that there is “virtue in slowness”; sub-diffusion, they posit, increases a particle’s probability of finding a nearby target. This is, perhaps, a metaphor for our scientific endeavours.

There is the exquisite complexity involved also in the absorption of nanoparticles by the oral route due to the dimensionally constraining and moving intestinal villi and microvilli and through their actin substructures. There are, too, the general issues of particokinetics [7] and three processes in target cell uptake: (i) endocytosis, (ii) diffusion of particles through cells and (iii) the potential “road block” of exocytosis [8]. All are of vital importance in reaching the exact narrative of nanoparticle delivery that we must embrace, critique and consider so that new approaches can be developed to surmount these difficulties. What of course might be applicable to biodegradable polymer particles may not apply to all systems such as dendrimers or carbon nanotubes. Such is the variety of carriers collective endeavours have produced.

## Debate

In the 1990s, de Gennes [9] mused on the hopes and illusions surrounding the field and it has been affirmed that “there are issues to be addressed everywhere: taming Brownian motion, working with weak finicky interactions, and developing a robust theoretical framework.” [10]**.** Lammers and Ferrari [11] have discussed successes in these endeavours; Park [12] has called for cessation of the hype that has surrounded the topic, examples including press releases describing nanosystems delivering “anticancer cluster bombs”.^1^ Critical questioning and appraisal is vital for the future health of the science. Couvreur [13] asserts that the nanomedicine field has reached the “slope of enlightenment.”

## The active molecules in carrier systems

The intrinsic potency, off-target toxicity [14] and specificity of the drugs we deliver are not always rigorously reported. Academic laboratories may not have ready access to new, more specific and effective agents. It has been emphasised [15] that in the wider context, small, as opposed to large, drug molecules can potentially bind to a wider range of extracellular and intercellular targets. There is also the vital importance of the physicochemical properties of the drugs we work with. Carriers perhaps cannot always be optimised for the optimal drug load, or to deliver actives at *appropriate rates* at *essential points* in their trajectory. Are multilayered drug systems needed? There are of course now physical methods to achieve drug release at certain points [16], but how much depends on the carriers having reached the required destination at the same time? Everything counts: size, shape, charge, flexibility, the material forming the nanosystems, drug loading and solubility in the carrier and drug behaviour in the target medium. Processes of aggregation, adsorption and engulfment by cells must be considered even in in vitro test systems. Higuchi’s [17] classic equation showed that the amount of drug released from a matrix is a function of the compound’s diffusion coefficient, its content in the matrix, the matrix porosity and drug solubility in the medium. This is applicable to most nanocarriers, but it is perhaps impossible to determine sink conditions at target sites. Even so, Siepmann and Siepmann [18] have emphasised that such external sink conditions do not guarantee the absence of saturation effects, as such may be formed *within* a delivery system. There is also the need to explore the influence on absorption of certain particle components such as lipids in liposomes or nonionic surfactants forming niosomes. One nonionic surfactant, Brij 30, enhances the activity of doxorubicin [19]. The singularity of dendrimers for example lies in their “open” chemical structures and thus their mode of carrying and releasing their loads may deviate from any norms. The quest must be for novel systems avoiding the deficiencies of many present constructs.

## Recognising complexity as an opportunity

Those, like the author, who began their research in the mid-1960s, were probing the field of drug delivery and learned slowly and imperfectly, partly because of the lack of the sophisticated instrumentation and techniques available today. Paradoxically, one of the problems today resides in the explosion of the literature, which often leads sadly to ignoring the older literature in adjacent or analogous fields. Our work on the oral delivery of nanoparticles [20] resulted largely from the stimulus of reading Verzár and McDougall’s 1936 book on *Absorption from the Intestine* [21]*.*

**Fig. 1:**
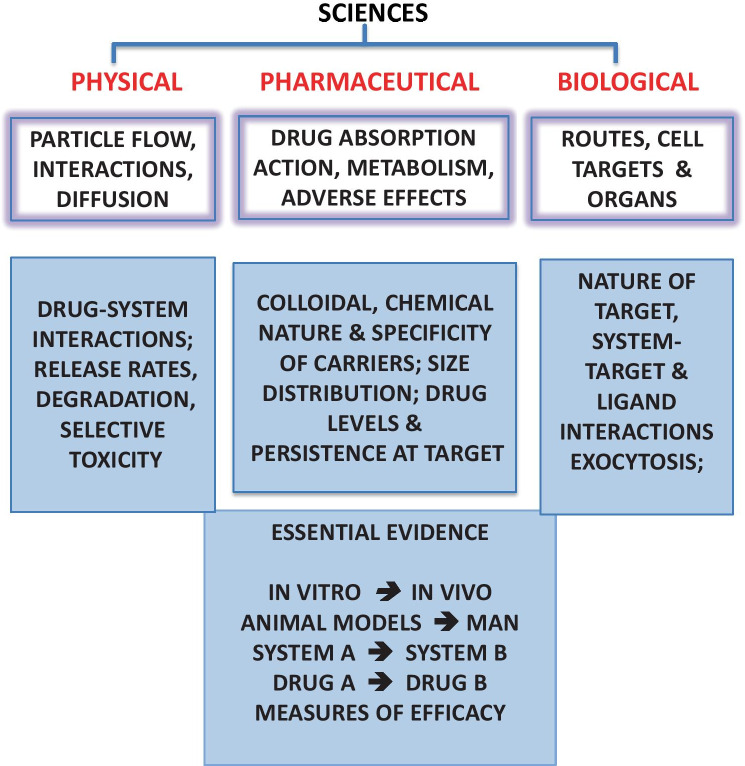
The many aspects of the sources of basic knowledge and their scope which include aspects of drugs, carriers and targets allowing the evaluation of personal results from experiments, including the nature of drug, carrier, target, critical reading of the scientific literature, serendipity and the consideration of analogies (see Fig. 2). In vitro data, valuable to highlight behaviour in controlled environments aids extrapolation to in vivo conditions. Animal data must be considered with comparisons of other systems and active agents to determine their true significance in man, aided by physical and mathematical modelling

Figure 1 depicts the sources and estuaries of knowledge in the field. Imagining simplicity serves no purpose. Grizzi et al. [22] even refer to “unsimplifiable complexity.”

Tumour targets may not only be heterogeneous, but may change their nature and structure as they grow. Targets can thus be dynamic systems, and it has been said [23] that a “tumour lives to change” and that we sometimes must attack changing or moving targets such as metastasized cancer cells [24]. The sequence and order of barriers must be acknowledged and the rate-limiting steps determined as part of research endeavours.

Exploring complexity is as an opportunity to define our future work searching for the influence of the diversity of nanosystems, their size, shape, flexibility, chemical nature, capacity, degradation, toxicity, the diversity of active agents and the nature of targets *inter alia.*

## Unexpected findings: serendipity

Finding the inexplicable in gathered data does not mean failure. This generally means that our postulates of potential outcomes have been incorrect or oversimplified. Experiments are but steps in determining reality. The unpredictable nature of many events in the passage and action of nanocarriers is a source of opportunity, as it provides the chance to discover novel aspects of our systems which may lead to new research avenues. Two examples include finding that a dendrimer we synthesised possessed intrinsic fluorescence [25], allowing analysis of its diffusional trajectory in cells, the other that a cationic polylysine dendrimer formed a complex with heparin, conferring on it anti-angiogenic and hence anticancer activity [26].

## Analogies

**Fig. 2:**
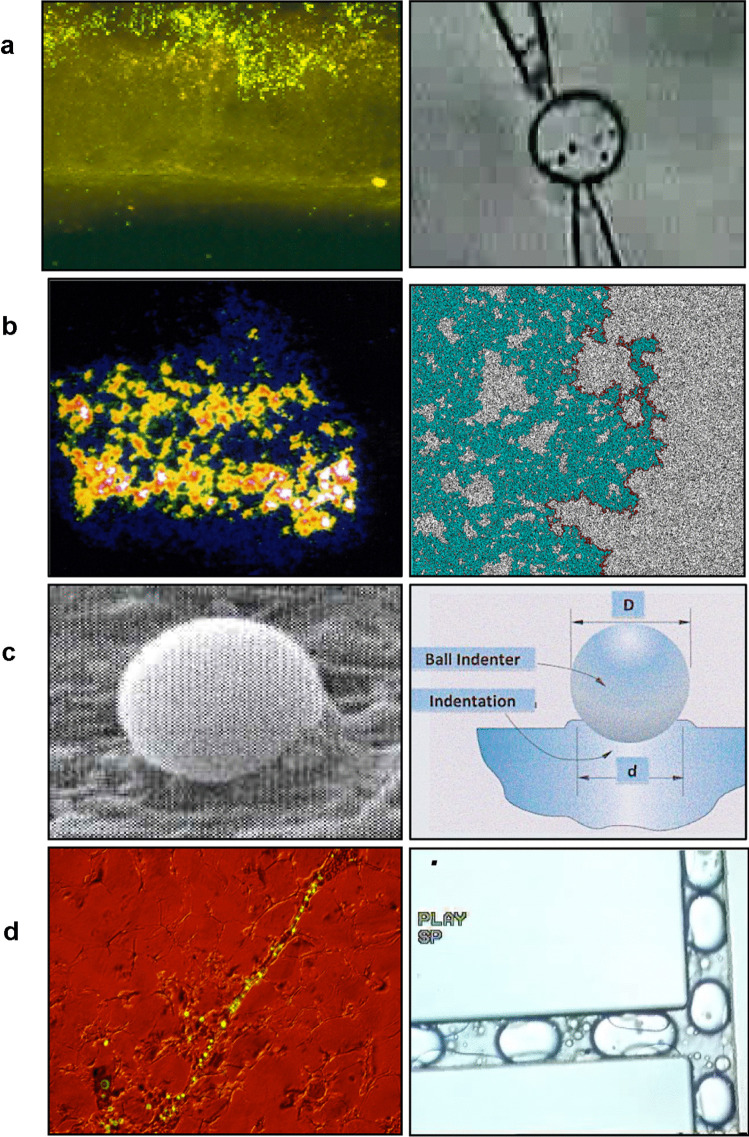
Examples of putative analogous systems for aspects of nanoparticle behaviour: **a** crowded nanoparticle environments and movement**:** left: nanoparticles at an intestinal surface; right: particles in a lipid vesicle to determine the effect of numbers and proximity on diffusion coefficients. **b** Uneven distribution of nanosystems: Left***:*** in a tumour spheroid (which could be considered a tumour analogue; right: a porous system for investigating particle suspension movement and penetration. **c** Factors in particle uptake: left: particle coated with invasin entering a cell monolayer; right: system used to determine particle-substrate interactions. **d** Particle flow. Left: nanoparticles in lymph vessels; right: movement of droplets in microfluidic systems to better understand flow behaviour

Edward de Bono [27] wrote that “creativity involves breaking out of established patterns in order to look at things in a different way.” The long history of colloids and their nano-offspring has made all aware of analogies between the physical and the biological fields. The sporadic motion of pollen on still water discovered by the botanist Robert Brown led Einstein to develop the theory of “Brownian” motion. Chu [28] has discussed laser light slowing down atoms using the analogy with Brownian motion of particles in a liquid. The cytoplasm of living cells has been said to be analogous to a poroelastic material [29] emphasizing the importance of cytoplasmic rheology in drug and nanoparticle access. Analogies are said to be useful for understanding sub-microscopic levels of reality [30]. Figure 2 gives some examples.

Currently, there is the relevant viral–nanoparticle analogy and we can ask to what extent viral particle trajectories are akin to those of nanoparticles? It was in 1937 that Elford [31] published work on the passage of viruses through membrane filters, so relevant today to determining the efficacy of facial masks. Sun and Wirtz [32] and Frey et al. [33] have discussed the similarities between nanoparticle and viral uptake into cells. The exigencies of the Covid-19 outbreak has stimulated research on nano-based disinfectants such as TiO_2_ [34] and dexamethasone nanomedicines [35]. These are examples of how new challenges can draw on existing knowledge and techniques in other domains. Taking advantage of the biological processes used for cell entry is a form of learning from nature, exemplified in utilizing bacterial mechanisms to enhance particle epithelial cell entry using invasin [36] or internalin fragments [37].

## Considerations and generalisations

The relationship between delivery of nanoparticles and issues surrounding the toxicity of particulate materials is clearly one of importance, raising the question whether nanomaterials “necessitate a new toxicological science” [38]. Carbon nanotubes have been considered to consist of a material which should be banned because of toxicity [39], but this has been rightly challenged on the grounds that not all carbon nanotubes are identical [40]. Generalisations are unscientific whether they refer to action, efficacy or toxicity. The effect of microparticles on the initiation of Crohn’s disease, such as from toothpastes containing titanium dioxide, has been discussed [41, 42]; having studied the oral uptake of titanium dioxide particles enabled our participation in the debate [43] about their potential harm. FDA trials are reported to have found in blood particulate ingredients of sunscreen products [44] generally regarded as “safe and effective.” Topical absorption here is relevant.

Wherever we are on our own trajectories, there are challenges in the work we do. Medawar [45] once said that science is “the art of the soluble” but we will rarely solve *all* the riddles with which we come face to face! The more we delve, the more we find.

## Outputs

How should we convey our scientific narratives? Science attempts to find the truth hence papers written as a result of any work must be totally open [46], never selective in publishing only data which give a semblance of “success.” Negative results are important so that the literature is not biased towards the positive. Titles of publications are occasionally used by authors to gain undue attention. With my own background synthesising non-ionic surfactants with micellar diameters in the 5–10 nm range, I ask now not only what are “nanomicelles” but question the added nonsense of “ultra small nanomicelles”? [47]. We all should be perplexed by the “immense uptake” of insulin by the oral route [48]. Papers should, rather, excite by introducing new concepts, new ways of looking at or identifying new issues. Examples I have been drawn to include the strange but evocative phrases such as the “discovery of slowness” [6] or “lateral diffusion in an archipelago” [49] which conjure up analogies with nature, and the physical concepts of advection, convection, obstruction and the like which add to the intriguing nature of the field of nanotechnology discussed here.

## Conclusion

Nanotechnology of course encompasses much more than drug delivery and its potential use in cancer therapy, but in all areas, the use of the prefix “nano” is a definition only of size range and not of potency. What path researchers take at the start of their careers matters, but the route thereafter is as vital. The poet Robert Frost wrote about the paths in life we take, himself reflecting that for him “the one less travelled by…. has made all the difference”. The true value of a scientific quest, writes Philip Ball [50] “comes from the journey, not the goal.” We need to explore uncrowded avenues, new directions surrounding all aspects of this intriguing field, including the nature of our targets. A recent paper [51] stressing the links between physics and biology discusses issues such as tumour stress, fluid pressure, stiffness and microarchitecture. Each day brings a new perspective!

## Footnotes

1 Emory Health Sciences. 29th March 2016.

## References

[1] Halbert GW, Stuart JFB, Florence AT (1985). A low density lipoprotein-methotrexate covalent complex and its activity against L1210 cells in vitro. Cancer Chemother Pharmacol.

[2] Maeda H (2015). Toward a full understanding of the EPR effect in primary and metastatic tumours as well as issues related to its heterogeneity. Adv Drug Del Rev.

[3] Wang JH (1954). Theory of the self-diffusion of water in protein solutions. A new method for studying the hydrodynamics and shape of protein molecules. J Amer Chem Soc.

[4] Elworthy PH, Florence AT, Rahman A (1972). Conductivity of sodium chloride and potassium chloride in polymer solutions and the obstruction effect. J Phys Chem.

[5] Ruenraroengsak P, Al-Jamal KT, Hartell N, Braeckmans K, De Smedt SC, Florence AT. Cell uptake, cytoplasmic diffusion and nuclear access of a 6.5nm diameter dendrimer. Int J Pharm. 2007;331:213–219. 10.1016/j.ijpharm.2006.12.012 17234370

[6] Guigas G, Weiss M (2008). Sampling the cell with anomalous diffusion- the discovery of slowness. Biophys J.

[7] Teeguarden PG, Hinderliter PM, Orr G, Thrall BD, Pounds JG (2007). Particokinetics in vitro: dosimetry considerations for in vitro nanoparticle toxicity assessments. Toxicol Sci.

[8] Dahiya UR, Ganguli M (2019). Exocytosis – a putative road block in nanoparticle and nanocomplex mediated gene delivery. J Control Rel.

[9] de-Gennes PG (1998). Nanoparticles and dendrimers: hopes and illusions. Croatia Chemica Acta.

[10] Editorial N (2019). Nanotech.

[11] Lammers T, Ferrari M (2020). The success of nanomedicine. Nano Today..

[12] Park K (2019). The beginning of the end of the nanomedicine hype. J Control Rel.

[13] Couvreur P (2019). Nanomedicine: from where are we coming and where are we going?. J Control Rel..

[14] Lin A, Guiliano CJ, Palladino et al. Off-target toxicity is a common mechanism of action of cancer drugs undergoing clinical trials. Sci Trans Med. 2019; 11. 10.1126/scitransmed.aaw841. 10.1126/scitranslmed.aaw8412 PMC7717492 31511426

[15] Bedard PL, Hyman DM, Davids MS, Siu LL (2020). Small molecules, big impact: 20 years of targeted therapy in oncology. Lancet.

[16] Wang F, Yuan, McMullen P, Li R, Zheng J (2019). Near-Infra-red-light responsive lipid nanoparticles as an intelligent drug release system for cancer therapy. Chem Mater.

[17] Higuchi T (1963). Mechanism of sustained-action medication. Theoretical analysis of rate of release of solid drug dispersed in solid matrices. J Pharm Sci.

[18] Siepmann J, Siepmann F (2020). Sink conditions do not guarantee the absence of saturation effects. Int J Pharm..

[19] Kerr DJ, Wheldon TE, Russell JG, Maurer HR, Florence AT (1987). The effect of the nonionic surfactant Brij 30 on the cytotoxicity of Adriamycin in monolayer, spheroid and clonogenic culture systems. Eur J Cancer Clin Oncol..

[20] Jani P, Halbert GW, Langridge J, Florence AT (1989). The uptake and translocation of latex nanospheres and microspheres after oral administration to rats. J Pharm Pharmacol.

[21] Verzar F, McDougall EJ (1936). Absorption from the Intestine.

[22] Grizzi F, Di Ieva A, Russo C, Frezza E E, et al. Cancer initiation and progression: an unsimplifiable complexity. Theor Biol Med Mod, 2006; 3, 37. 10.1186/1742-4682-3-37. 10.1186/1742-4682-3-37 PMC1621057 17044918

[23] Chaplain MJ (2009). Preface. J Math Biol.

[24] Li J, Ai Y, Bu P, Sharkey CC (2016). Targeted drug delivery to circulating cells via platelet membrane-functionalized particles. Biomaterials.

[25] Al-Jamal KT, Al-Jamal WT, Kosterelos K, Turton JA, Florence AT (2012). Anti-angiogenic Poly-l-lysine dendrimer binds heparin and neutralises it activity. Results Pharm Sci.

[26] Al-Jamal KT, Al-Jamal WT, Akerman S, Podesta JE, Yilmazer A, Turton JA (2010). Systemic antiangiogenic activity of cationic poly-L-lysine dendrimer delays tumor growth. Proc Natl Acad Sci USA.

[27] De Bono, E. Lateral Thinking for Management. See debono.com.

[28] Chu S (1998). Nobel Lecture. The manipulation of neutral particles. Rev Mod Phys.

[29] Moeendarbary E, Valon L, Fritzsche M (2013). The cytoplasm of living cells behaves as a poroelastic material. Nat Mat.

[30] Del Re G (2000). Models in analogies in science. Int J Phil Chem.

[31] Elford WJ (1937). Principles governing the preparation of membranes having graded porosities. The properties of “Gradacol” membranes as ultrafilters. Trans Farad Soc.

[32] Sun XS, Wirtz D (2006). Mechanics of enveloped virus entry into host cells. Biophys J..

[33] Frey F, Ziebert F, Schwarz US (2019). Stochastic dynamics of nanoparticle and virus uptake. Phys Rev Lett.

[34] Talebian S, Wallace GG, Schroeder A, Stellaci F, Conde J (2020). Nanotechnology-based disinfectants and sensors for SARS-CoV-2. Nat Nanotech.

[35] Lammers T, Sofias AM, van der Meel R, Schiffelers R (2020). Dexamethasone nanomedicines for Covid-19. Nat Nanotech..

[36] Hussain N, Florence AT (1998). Utilizing bacterial mechanisms of epithelial cell entry: invasin-induced oral uptake. Pharm Res.

[37] Roland R E S , Taylor P W, Florence A T. Attachment, uptake, transport of nanoparticles coated with an internalin A fragment in CacO_2_ cell monolayers. J Drug Del Sci Tech; 2005; 313–17

[38] Nel A, Xia T, Mädler L, Li N (2006). Toxic potential of materials at the nanolevel. Science.

[39] Hansen SF, Lennquist A (2020). Carbon nanotubes added to the SIN List as a nanomaterial of very high concern. Nat Nanotech.

[40] Fadeel B, Kostarelos K (2020). Grouping all carbon nanotubes into a single substance category is scientifically unjustified. Nat Nanotech.

[41] Sullivan SN (1990). Hypothesis revisited: toothpaste and the cause of Crohn’s disease. Lancet.

[42] Powell JJ, Harvey RS, Thompson RPH (1996). Microparticles in Crohn’s disease-has the dust settled?. Gut.

[43] Florence AT, Jani PU, McCarthy D (1990). Toothpaste and Crohn’s disease. Lancet.

[44] Abbasi J. FDA trials find sunscreen ingredients in blood but risk is uncertain. JAMA, 2020; 325:1431-2 10.1001/jama.2020.0792 32236495

[45] Medawar PB (1969). The Art of the Soluble.

[46] Florence AT (2017). Style and precision in scientific papers. Int J Pharm.

[47]  Song K,  Xin M, Zhang F (2020). Novel ultrasmall nanomicelles based on rebaudioside A: a potential nanoplatform for the ocular delivery of pterostilbene. Int. J Pharm.

[48] Kim KS, Kwag DS, Hwang HS (2018). Immense insulin intestinal uptake and lymphatic transport using bile acid conjugated partially uncapped liposome. Mol Pharm.

[49] Saxton MJ (1993). Lateral diffusion in an archipelago. Biophys J.

[50] Ball P. The holy grail myth. Chemistry World. 2020;17(10):43.

[51] Nia HT, Munn LL, Jain RK (2020). Physical traits of cancer. Science.

